# Detecting Potential Adverse Reactions of Sulpiride in Schizophrenic Patients by Prescription Sequence Symmetry Analysis

**DOI:** 10.1371/journal.pone.0089795

**Published:** 2014-02-27

**Authors:** Edward Chia-Cheng Lai, Cheng-Yang Hsieh, Yea-Huei Kao Yang, Swu-Jane Lin

**Affiliations:** 1 Institute of Clinical Pharmacy and Pharmaceutical Sciences, National Cheng-Kung University, Tainan, Taiwan; 2 Department of Neurology, Tainan Sin-Lau Hospital, Tainan, Taiwan; 3 Health Outcome Research Center, National Cheng-Kung University, Tainan, Taiwan; 4 Department of Pharmacy Administration, College of Pharmacy, University of Illinois at Chicago, Chicago, Illinois, United States of America; Baylor College of Medicine, United States of America

## Abstract

**Purpose:**

Previous studies have demonstrated sulpiride to be significantly more effective than haloperidol, risperidone and olanzapine in schizophrenic treatment; however, only limited information is available on the potential risks associated with sulpiride treatment. This study attempts to provide information on the potential risks of sulpiride treatment of schizophrenia, especially with regard to unexpected adverse effects.

**Materials and Methods:**

Patients with schizophrenia aged 18 and older, newly prescribed with a single antipsychotic medication from the National Health Insurance Research Database of Taiwan in the period from 2003 to 2010 were included. A within-subject comparison method, prescription sequence symmetry analysis (PSSA) was employed to efficiently identify potential causal relationships while controlling for potential selection bias.

**Results:**

A total of 5,750 patients, with a mean age of 39, approximately half of whom were male, constituted the study cohort. The PSSA found that sulpiride was associated with EPS (adjusted SR, 1.73; 95% CI, 1.46–2.06) and hyperprolactinemia (12.04; 1.59–91.2). In comparison, EPS caused by haloperidol has a magnitude of 1.99 when analyzed with PSSA, and hyperprolactinemia caused by amisulpride has a magnitude of 8.05, respectively. Another finding was the unexpected increase in the use of stomatological corticosteroids, emollient laxatives, dermatological preparations of corticosteroids, quinolone antibacterials, and topical products for joint and muscular pain, after initiation of sulpiride treatment.

**Conclusions:**

We found sulpiride to be associated with an increased risk of EPS and hyperprolactinemia, and the potential risk could be as high as that induced by haloperidol and amisulpride, respectively. Additionally, our study provides grounds for future investigations into the associations between sulpiride and the increased use of additional drugs for managing adverse effects, including stomatological, dermatological, and musculoskeletal or joint side effects, constipation, and pneumonia.

## Introduction

The number of currently available antipsychotic medications, each with unique effectiveness and side effect profile, has made it feasible to individualize regimen to achieve optimal antipsychotic therapy and this has now become standard practice for patients with schizophrenia [1]. Optimal antipsychotic therapy requires a psychiatrist to select a viable regimen based on global assessment of individual patients by weighing safety and tolerability of drugs against their efficacy [1], [2]. Adverse events (AEs) induced by antipsychotics could significantly impede a patient’s adherence to treatment and in turn diminish the therapeutic benefit, potentially reducing health and quality of life (e.g., movement disorder due to dopamine blockade) [3]. Understanding risks of antipsychotics is essential for managing unintended outcomes and achieving successful treatment [1].

Novel antipsychotics, namely atypical antipsychotics (AA), have developed rapidly in recent decades. The consumption of AA, which are generally more expensive, has increased dramatically and thus generated considerable economic burden on the medical care system [4]. Previously, a comparative effectiveness study showed that sulpiride, a relatively affordable typical antipsychotic (TA), was significantly more effective than haloperidol, risperidone and olanzapine in treating schizophrenia, potentially providing a cost-effective alternative to the more expensive AAs and curbing the high and rising cost of antipsychotic treatment [5]. However, the limited information on sulpiride associated AEs in the literature might impact its adoption. Even though sulpiride has been widely used in some European and Asian countries for decades, only a handful of studies involving the drug have been conducted [6]–[9]. Clinical trials that explored sulpiride were limited by the relatively smaller sample size and the lack of generalizability [8], [10]. There were no data for many important outcomes concerning adverse effects of sulpiride.

Due to limitations of previous studies on sulpiride associated AEs, it is difficult for physicians to determine the role of sulpiride in clinical therapy and for decision makers to evaluate true costs of this medication. Using a large nationwide database, this study attempts to identify and estimate the magnitude of sulpiride associated AE risks. The risks of sulpiride in patients with schizophrenia were compared with other TAs (e.g., haloperidol) and AAs (e.g., risperidone). This study analyzed AEs that have been associated with antipsychotics and also comprehensively investigated potential AEs related to sulpiride use that have not yet to be detected or reported. PSSA was used to examine the distribution of marker drugs (potentially used for managing AEs), before and after initiation of sulpiride treatment, where in increased in the use of marker drugs after sulpiride might indicate an increase in AEs associated with sulpiride treatment.

## Methods

### Data Source

Electronic datasets for this study were obtained from the National Health Insurance Research Database (NHIRD) in Taiwan [11], maintained and made accessible for research purposes by the National Health Research Institute (NHRI). Taiwan launched a single-payer and mandatory National Health Insurance program on March 1, 1995, and by 2011, the entire Taiwan population (approximately 23.16 million individuals) was all enrolled. The NHRI compiles information on enrollees' demographics, health care professionals and facilities, service claims from inpatient, ambulatory care, and contracted pharmacies for reimbursement purposes. Personal identities are encrypted for privacy protection, but all data sets can be linked by unique, anonymous identifiers created by NHRI. Using NHIRD without cross linkage to other health data is exempt from ethical review in Taiwan. All the antipsychotics and most prescription drugs have been reimbursed by NHI in Taiwan, and all the records of reimbursed drug from inpatient, outpatient, and emergency service, and contracted pharmacies settings were included in NHIRD. Accuracy of major disease diagnoses in the NHIRD, such as stroke, epilepsy, and acute coronary syndrome, has been validated [12]–[14]. We used 3 Longitudinal Health Insurance Databases established by the NHRI; LHID2000, LHID2005, and LHID2010, each containing a cohort of 1 million beneficiaries, randomly sampled from the year 2000, 2005, and 2010 registries. Chi-square tests at alpha level 0.05 revealed no significant differences in distribution of age, gender, annual births, and average premium paid, between patients in sampled databases and the original NHIRD. Details of the sampling process are published online by Taiwan National Health Research Institutes [11].

### Ethics Statement

The study was approved by the Institutional Review Board of National Cheng-Kung Univisity. All researchers signed a written agreement declaring that they have no intention of attempting to obtain information from NHIRD that could potentially violate the privacy of patients or care providers.

### Study Design

Prescription sequence symmetry analysis, or PSSA, by *Hallas* [*15*] is an effective surveillance tool for drug associated AEs [16]. Because PSSA is based on within-subject comparison, the method allows patients to serve as their own comparator, similar to the case-crossover design suggested by Maclure [17], where exposures during a fixed period before case dates (dates when the target outcomes happened) and some prior dates in the same individual were compared. These within-subject comparisons can thus be fully controlled for potential confounding from between-subject differences and time-invariant characteristics, e.g., age, gender, genetic factors, mental health status, polypharmacy, and other unknown confounding factors. PSSA has also been employed in previous studies investigating associations between use of certain target drugs and potential AEs such as depression [15], hip fracture [18], or nocturnal leg cramps [19]. The validity of the PSSA has been confirmed by previous study [20], [21].

PSSA was performed in this study to explore association between antipsychotics exposure and related AEs. Briefly, it tests the propensity to initiate a marker drug (e.g., trihexyphenidyl) after use of an index drug (e.g., antipsychotics), where the index drug is suspected inducing a side effect (e.g., extrapyramidal symptom) that warrants treatment with the marker drug [22]. Theoretically, if there is no causal relationship between index and marker drugs, it is equally possible for patients to initiate the marker drug before or after index drug initiation; resulting in a symmetrical (or random) prescribing pattern of the marker drug around index drug initiation. Conversely, if the index drug increases the risk of an AE requiring treatment with the marker drug, it is expected that the marker drug is more likely to be initiated after rather than before the index drug, leading to an asymmetrical marker drug prescribing pattern.

### Study Cohort and PSSA Selection

This study identified a cohort of patients with schizophrenia by International Classification of Diseases, Ninth Revision, Clinical Modification (ICD-9-CM) code 295.XX, between 2003 and 2010, who were aged 18 or older, and new to single antipsychotic drug treatment, including sulpiride, haloperidol, olanzapine, quetiapine, amisulpride, risperidone, or aripiprazole. We considered the first prescribed antipsychotic drug after diagnosis as the index drug, and its first prescription date the index date. Antipsychotic users who had not received any antipsychotic prescription during 6 years prior to the index date were considered new users. Patients without 6 years of NHI eligibility prior to index date were excluded to ensure sufficient data to identify new user status.

**Figure 1:**
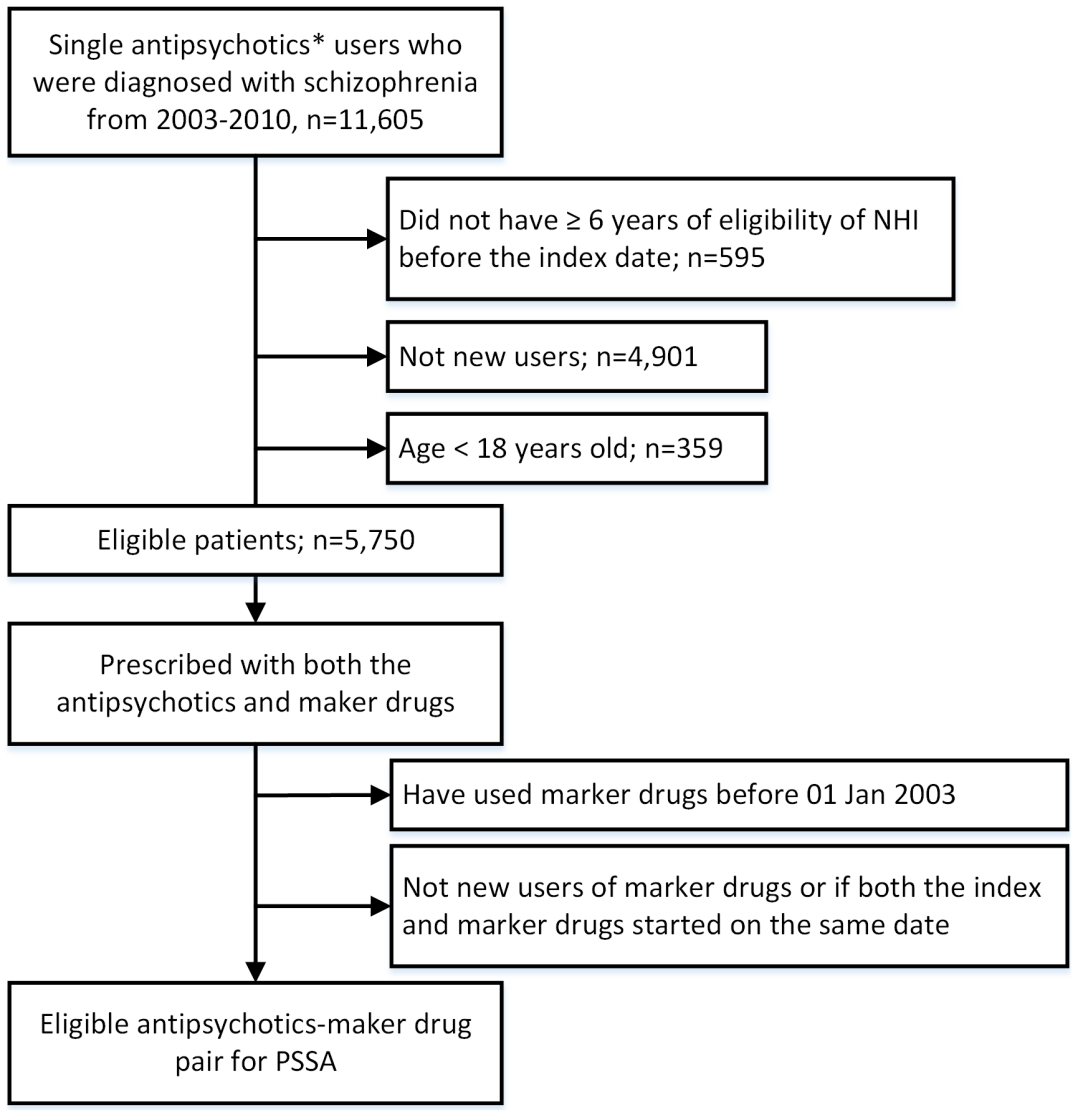
Flowchart of study cohort selection. Footnote of figure1: PSSA: Prescription Sequence Symmetry Analysis; NHI: national health insurance. *Include sulpiride, haloperidol, risperidone, olanzapine, quetiapine, and amisulpride.

A 12-month “waiting time” was imposed as a baseline period to ensure a marker drug (e.g., trihexyphenidyl used to manage extrapyramidal syndromes of haloperidol) was indeed newly prescribed. In other words, a marker drug was considered a new prescription if not used in the previous 12 months. Hence, the earliest possible marker drug prescription date was January 1 2003. Baseline characteristics were extracted from records within one year prior to the index date. The observation period for sequences of incident marker drug use was restricted to 12 months before and after incident index drug use to reduce potential impacts from within-subject confounding, such as maturation and other potential time-varying covariates (e.g., dietary change) that could occur in a long study period. Figure 1 shows the study cohort selection process.

Two sets of PSSA were carried out; one confirmatory in nature where AEs reported in the literature were examined; the other exploratory, to identify potential sulpiride associated AEs in schizophrenia patients.

### Confirmatory analyses

We performed confirmatory analyses to extend current evidence and tested associations between sulpiride (index drug) and AEs suggested or confirmed in other antipsychotics, including (1) extrapyramidal syndromes (EPS), (2) metabolic syndrome such as hyperglycemia, (3) hyperprolactinemia, and (4) cardiac arrhythmias. Corresponding drug treatments intended to manage aforementioned AEs were used as marker drugs, including anticholinergic agents for EPS (i.e., trihexyphenidyl), oral hyperglycemic agent for hyperglycemia, prolactine inhibitors for managing hyperprolactinemia, and class 1B antiarrhythmic agents for cardiac arrhythmias. Other non-sulpiride antipsychotics, including haloperidol, risperidone, olanzapine, quetiapine, and amisulpride were used as references and included in the analyses for two purposes: to benchmark AE risk magnitude between antipsychotics and to check validity of PSSA results by comparing with previously published studies.

### Exploratory analyses

Since publications on sulpiride AEs are limited, some less anticipated AEs might have been overlooked. Thus, in part two of this study, we performed exploratory analyses to identify AEs previously not considered sulpiride associated in patients with schizophrenia. Firstly, all medications prescribed after the index date were considered candidate marker drugs to treat AEs. These marker drugs were classified by the Anatomical Therapeutic Chemical (ATC) classification system [23] developed by the WHO Collaborating Centre, which divided all substances into different subgroups according to organ or system acted upon, and therapeutic and chemical properties. In the first part, marker drugs were classified into pharmacological subgroups following the 4-digit level of the ATC codes; that is, from class A01A (i.e., stomatological preparations) to class S03D (i.e., other ophthalmological and otological preparations), and then tested by PSSA for sulpiride associations. Nervous system drugs, from class N01A (anesthetics) to N06D (anti-dementia drugs), were excluded because antipsychotics’ effects on the nervous system cannot easily be separated from those of other underlying psychiatric disease. Candidate pharmacological subgroups of marker drugs that reached statistical significance at alpha level 0.1 were then re-classified by chemical subgroup following the 5-digit level of the ATC codes and again tested for sulpiride association.

These exploratory analyses were based directly on prescribed medications without specifically considering their roles in handling AEs for three reasons: (1) when associations of certain AEs and antipsychotics have yet to be well established, diagnoses of these AEs may not be recognized and recorded by physicians, (2) because at most 5 diagnoses can be recorded in any single NHIRD record, secondary diagnoses associated with AEs may not be fully captured in the database leading to underestimation of AE risks, (3) a drug prescription may imply an AE was clinically significant, requiring intervention by a countermeasure.

### Statistical Analysis

The ratio of patients initiating a marker drug after the index drug (index→marker) to those initiating a marker drug before the index drug (marker→index) is defined as the crude sequence ratio (SR). Although PSSA and SR help to minimize potential differences in baseline characteristics or those unrecognized confounding factors possibly present in between-subject comparisons, PSSA could be sensitive to prescribing trends (e.g., rapid increase in marker drug use) over time. For this reason, the null-effect SR (*SR_null_*) was calculated to adjust for any possible temporal trends. *SR_null_* is the expected SR of an incidence trend when there is no causal relationship between index and marker drugs, providing a background rate for the chronological sequence of two drugs [15]. In this study, we computed the probability of index to marker drug sequence for each user of an antipsychotic drug, at first prescription. The overall probability of antipsychotics use, *P_a_*, was generated by weighting the number of incident users on each prescribing date of antipsychotics and averaging over all days. The *SR_null_* was then computed as *P_a_*/(1– *P_a_*). Details of *SR_null_* computation are described in the **File S1**. The adjusted SR was derived by dividing crude SR with *SR_null_*, and 95% confidence intervals (CI) were determined with a normal approximation to the binomial distribution [24]. Significance at an alpha level 0.05 indicated a relevant causality between antipsychotics and defined AEs. All analyses were performed using SAS version 9.2 (SAS Institute, Inc., Cary, NC).

## Results

**Figure 2:**
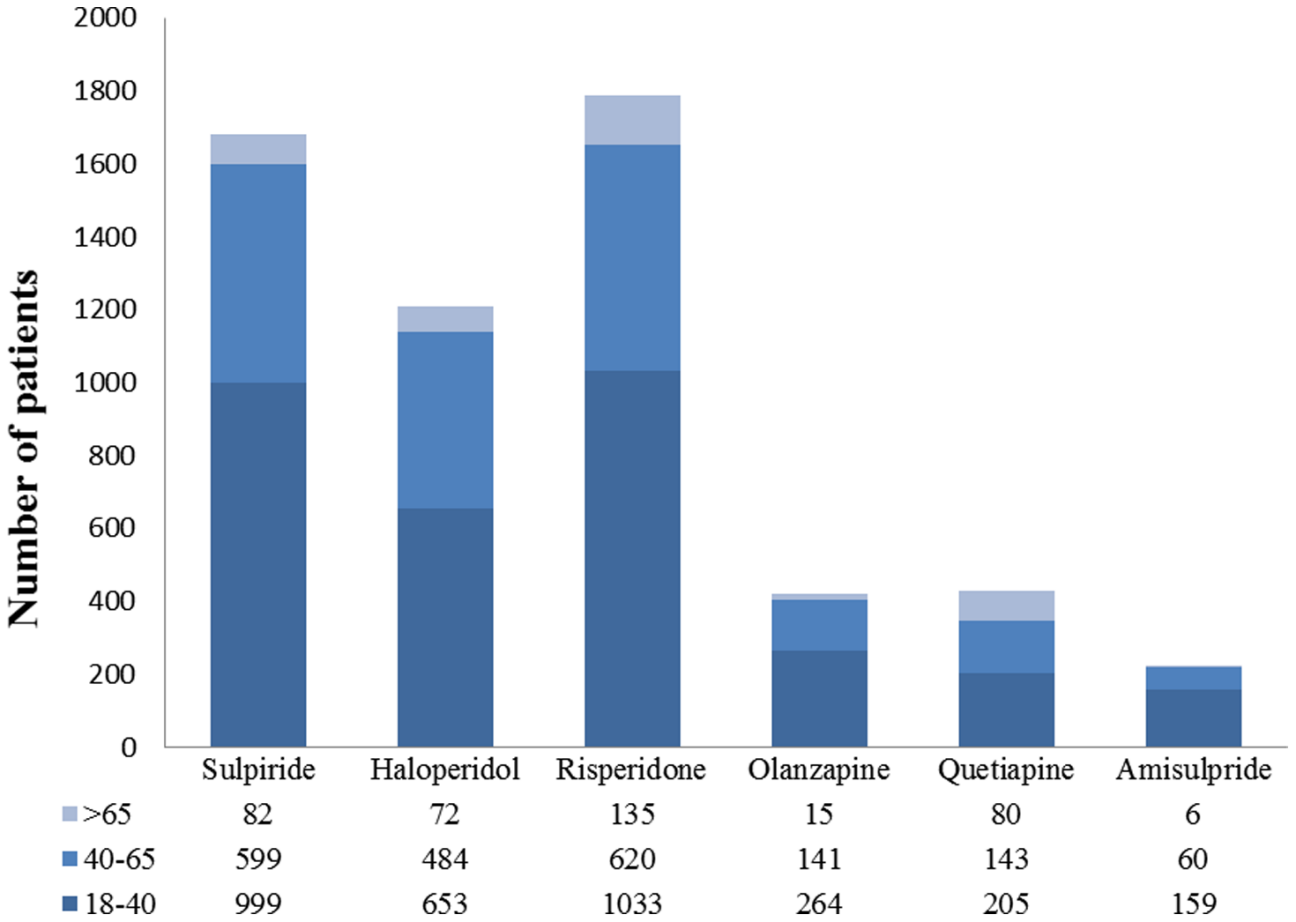
The distribution of age of patients by antipsychotic drugs.

From a population of 3 million patients, 11,605 with schizophrenia were identified who had used one of the study antipsychotics. We excluded 595 patients without at least 6 years NHI eligibility prior to the index date. Also excluded were 4,901 patients who were not new users during the study period. In addition, 359 patients younger than 18 years old at index date were excluded. (**Figure 1**) A total of 5,750 patients, with a mean age of 39, constituted the study cohort, of which approximately half were male. The distribution of antipsychotics by patients’ age is shown in **Figure 2**. Age distribution was roughly similar in each drug group except for a higher proportion of elderly receiving quetiapine.

### Confirmatory analyses

**Figure 3:**
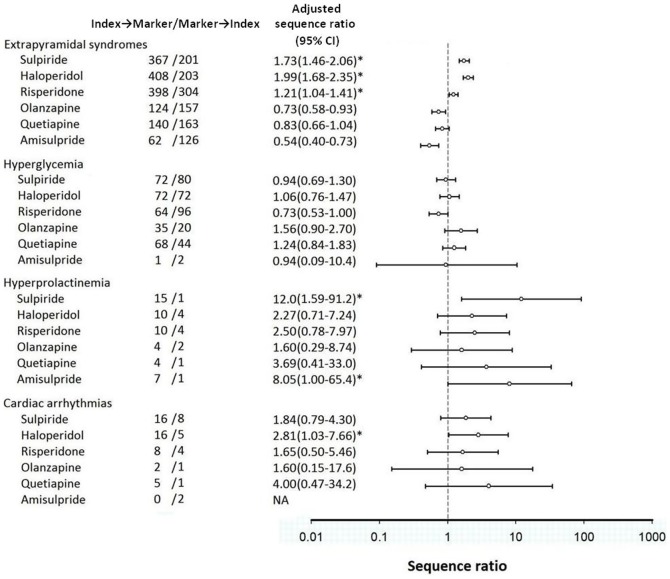
Confirmatory analyses on adverse events of sulpiride and other antipsychotics. Footnote of figure 3: NA: Not applicable, *Statistical significant at alpha level at 0.05.

Among the total of 1,680 incident sulpiride users, 568 had also been prescribed an anticholinergic agent, with 367 in the index→marker group(i.e., trihexyphenidyl prescribed after sulpiride) and 201 in the marker→index group (i.e., trihexyphenidyl started before sulpiride). The results of PSSA, based on anticholinergic use as a signal of EPS, found sulpiride significantly associated with EPS (adjusted SR: 1.73; 95% confidence interval 1.46–2.06), with risk only slightly lower than haloperidol (1.99; 1.68–2.35) but higher than risperidone (1.21; 1.04–1.41). Similarly, sulpiride was associated with hyperprolactinemia (12.04; 1.59–91.2) and cardiac arrhythmias (1.84; 0.79–4.30), although no statistical significance was found for cardiac arrhythmias. No association was found between sulpiride and hyperglycemia (0.94; 0.69–1.30). Antipsychotics with the highest effect size were haloperidol for EPS (1.99; 1.68–2.35) and cardiac arrhythmias (2.81; 1.03–7.66), olanzapine for hyperglycemia (1.56; 0.90–2.70), and amisulpride for hyperprolactinemia (8.05 ; 1.00–65.4). (**Figure 3**).

### Exploratory analyses

Eight classes of candidate marker drugs (based on 4 digit ATC codes) that might signal potential AEs after antipsychotic use were significant in exploratory analyses with PSSA, including stomatological preparations, laxatives, drugs for acid related disorders, blood and related products, beta blocking agents, dermatological preparations of combination formulations of corticosteroids, quinolone antibacterials, and topical products for joint and muscular pain. Details are shown in the **Table S1 in File S1**. Further analyses based on 5 digit ATC codes of drug groups belonging to the eight classes of significant candidate marker drugs showed a significant increase in use of the following drugs after sulpiride initiation: corticosteroids for local oral treatment (1.71; 1.00–2.91), emollient laxatives (1.55; 1.18–2.04), non-selective beta blocking agents (1.61; 1.28–2.03), weak potency combination formulations of corticosteroids (2.15; 1.08–4.28), fluoroquinolone antibacterials (1.81; 1.03–3.17), and non-steroid antiinflammatory preparations for topical use (1.36; 1.01–1.84). There was also an increased, but not statistically significant, use of drugs for acid related disorders (2.39; 0.89–6.43) and blood substitutes and plasma protein fractions (2.08; 0.92–4.72) after starting sulpiride. (**Table 1**).

**Table 1:** Exploratory analyses on possible adverse events of sulpiride by ATC groups.

Drug classification	ATC†	Index→Marker/Marker→Index‡			Sequence ratio		
					Crude	Adjusted	(95% CIs)
Stomatological preparations	A01A	48	/	23	2.09	1.86	(1.13–3.07)*
Caries prophylactic agents	A01AA	0	/	0	NA	NA	(. –. )
Antiinfectives and antiseptics for local oral treatment	A01AB	10	/	2	5.00	4.11	(0.90–18.8)
Corticosteroids for local oral treatment	A01AC	38	/	21	1.81	1.71	(1.00–2.91)*
Other agents for local oral treatment	A01AD	0	/	0	NA	NA	(. –. )
Other drugs for acid related disorders	A02X	18	/	5	3.60	2.39	(0.89–6.43)
Laxatives	A06A	181	/	110	1.65	1.44	(1.11–1.87)
Emollient softeners	A06AA	136	/	83	1.63	1.55	(1.18–2.04)
Contact laxatives	A06AB	16	/	10	1.63	1.57	(0.71–3.46)
Bulk producers	A06AC	29	/	17	1.59	1.48	(0.81–2.69)
Blood and related products	B05A	20	/	8	2.50	2.08	(0.92–4.72)
Blood substitutes and plasma protein fractions	B05AA	20	/	8	2.50	2.08	(0.92–4.72)
Other blood products	B05AX	0	/	0	NA	NA	
Beta blocking agents	C07A	225	/	146	1.54	1.42	(1.12–1.71)*
Beta blocking agents, non-selective	C07AA	190	/	114	1.67	1.61	(1.28–2.03)*
Beta blocking agents, selective	C07AB	24	/	42	0.57	0.56	(0.49–1.26)
Alpha and beta blocking agents	C07AG	11	/	11	1.00	0.98	(0.61–2.85)
Dermatological preparations, corticosteroids	D07X	42	/	15	2.80	2.18	(1.21–3.92)*
Corticosteroids, weak, other combinations	D07XA	31	/	11	2.82	2.15	(1.08–4.28)*
Corticosteroids, moderately potent, other combinations	D07XB	2	/	1	2.00	1.87	(0.42–30.6)
Corticosteroids, potent, other combinations	D07XC	9	/	3	3.00	2.30	(0.63–8.36)
Corticosteroids, very potent, other combinations	D07XD	0	/	0	NA	NA	(. –. )
Quinolone antibacterials	J01M	62	/	39	1.59	1.50	(1.00–2.24)*
Fluoroquinolones	J01MA	37	/	18	2.06	1.81	(1.03–3.17)*
Other quinolones	J01MB	25	/	21	1.19	1.17	(0.78–2.39)
Topical products for joint and muscular pain	M02A	103	/	77	1.34	1.31	(0.97–1.76)
Antiinflammatory preparations, non-steroidal for topical use	M02AA	100	/	73	1.37	1.36	(1.01–1.84)*
Capsaicin and similar agents	M02AB	0	/	0	NA	NA	(. –. )
Preparations with salicylic acid derivatives	M02AC	3	/	4	0.75	0.78	(0.17–3.49)
Other topical products for joint and muscular pain	M02AX	0	/	0	NA	NA	(. –. )

NA: Not applicable.

*Statistical significant at alpha level at 0.05.

† Anatomical Therapeutic Chemical (ATC) classification system developed by WHO Collaborating Centre.

‡ Index→Marker: patients initiating the marker drug after initiating the index drug; Marker→Index: patients initiating the index drug after initiating the marker drug.

## Discussion

Some AEs are predictable based on the action mechanism of a drug, for example, antipsychotics block dopamine receptors in the cortex, leading to EPS and movement disorders [22], [25], [26], dopamine blockade in the pituitary gland leads to hyperprolactinemia [27]. In the confirmatory analyses of the current study, we performed PSSA to evaluate sulpiride’s therapeutic risk; the results indicating sulpiride use was associated with significantly increased risks of EPS and hyperprolactinemia, with the magnitude of increased risk after sulpiride initiation only slightly lower than haloperidol but higher than risperidone. Furthermore, we carried out exploratory analyses in an attempt to identify less anticipated AEs where there are no clear or well-studied underlying pharmacophysiological mechanisms suggesting the association. From the exploration, we found sulpiride to be associated with increased use of several marker drugs, which might be used to manage some emergent AEs after sulpiride treatment.

Antipsychotic induced EPS is widely discussed in the literature, especially for AAs such as haloperidol. Our study found that the risks (adjusted SR) of EPS were 1.99, 1.21, 0.73, and 0.83 for haloperidol, risperidone, olanzapine, and quetiapine, respectively. If using haloperidol as reference drug, the relative risks of EPS are 0.61 in risperidone, 0.37 in olanzapine, and 0.42 in quetiapine. The results resemble a meta-analysis of 150 clinical studies conducted by Leucht et al. [25] where the authors found that, when compared to haloperidol, the relative risks of EPS were 0.61 in risperidone, 0.39 in olanzapine, and 0.43 in quetiapine. These results confirmed the validity of using PSSA in the current study to evaluate therapeutic risk of antipsychotics, and that sulpiride induced EPS risk (adjusted SR of 1.73) was higher than SGAs.

Metabolic syndrome such as hyperglycemia has been discussed by literature with the general conclusion that risk of metabolic syndrome from SGAs is higher than from haloperidol [25]. Rummel-Kluge et al. [28] included 54 randomized controlled trials and conducted head-to-head meta-analysis comparisons on risk of metabolic syndrome among SGAs. They found olanzapine more likely to increase cholesterol and glucose levels than other SGAs such as risperidone and quetiapine. Our results found that, compared to other SGAs, olanzapine had increased, though not statistically significant, probability of using hyperglycemic agents. The one-year observation period in our study, employed to reduce impacts from within-subject confounding, may be insufficient to capture certain AEs requiring longer duration to surface.

As a case-only study design, PSSA has the strength of being able to capture a signal event efficiently when investigating AEs with lower incident rate [17]. For example, PSSA can be used to identify those rare cases with markedly raised levels of prolactin after antipsychotics use, where corrective intervention with prolactin inhibitors (e.g., cabergoline) might be indicated to manage severe hyperprolactinemia [29]. In the current study, we did find some cases of hyperprolactinemia among patients receiving sulpiride and amisulpride [30], requiring prolactine inhibitor treatment, with high effect magnitudes. However, the detection bias may play a role when using PSSA since patients might be more likely to receive diagnostic procedure after, rather than before, the initiation of antipsychotics. Nevertheless, the effect of detection bias in this study might have been minimized after adjusting the prescribing trend or with reference to the PSSA pattern of other antipsychotics where the risk profile has been well-established (e.g., amisulpride and hyperprolactinemia) [29].

In the exploratory analyses, we found that sulpiride treatment increased the likelihood of oral corticosteroid use, which could be indicated for wounds in the mouth, such as aphthous ulcer, or other oral inflammation situations. Limited evidence is available about stomatological side effects induced by antipsychotics [31]. If it is indeed a sulpiride AE, the mechanism of action might be decreased salivary secretion from blockade of muscarinic receptors [32], or dental movement disorders from blockade of dopamine receptors [33]. One clinical trial reported that sulpiride induced oral ulcers led to treatment discontinuation [31]. It might be advisable to consider stomatological care in patients receiving sulpiride, to reduce risks of caries, gingivitis, periodontitis and stomatitis, and to prevent poor adherence and treatment failure [34]. The blockade of muscarinic receptors also led to some anticholinergic side effects such as constipation [32], as reflected by a significantly higher proportion of patients initiating emollient laxatives after sulpiride compared to before sulpiride. We found sulpiride use increased likelihood of using dermatological preparations of weak potency corticosteroids, possibly indicating clinical manifestations of skin pruritus or xerosis, and possibly resulting from anticholinergic effects of sulpiride on sweat glands [32]. Increased likelihood of non-selective beta-blocker indicates possible sulpiride induced tachycardia or hand tremor symptoms. However, non-selective beta-blockers (e.g., propranolol) could also be used as adjuvants in controlling symptomatic anxiety of patients with schizophrenia [35]. We considered the increased likelihood of topical use of nonsteroidal anti-inflammatory drugs (NSAIDs) as an indication of musculoskeletal or joint pain. Such troublesome AEs may decrease quality of life and lead to future noncompliance of patients [36]. Fluoroquinolone antibacterials are commonly used for community-acquired pneumonia, and its association with antipsychotics has been recently discussed in the literature [37]–[40]; however, these studies have not investigated the sulpiride role in infection. Our results indicated sulpiride increased the likelihood of fluoroquinolone use, potentially reflecting the risk of community-acquired pneumonia. Psychiatrists who start patients on sulpiride should closely monitor signs of pneumonia.

We have reviewed the literature and suggested a possible mechanism of action whereby sulpiride could cause aforementioned potential AEs, and the results of exploratory analyses could strengthen clinical awareness of psychiatrists when using sulpiride. However, we should bear in mind that many drugs were tested in exploratory analyses, which could increase the likelihood of type-1 error (false positive) where a significant association might actually be spurious. Further studies are needed to obtain more evidence on the potential sulpiride AEs.

The confirmatory analyses were based on published literature that such relationships have been observed. PSSA was used to evaluate the efficiency and statistical significance of using this method to detect the known relationships. There was no priori hypothesis for the exploratory analysis in the current study; the exploratory analysis focused on rapidly screening for yet to be detected drug-AE relationships. This part of the study did not imply a definite causality of the drug-AE association; it only suggests a statistical association demonstrated by the asymmetry chronological pattern of target drug and drugs used to manage AEs. As to whether the association is also causality, it will depend on future study design that can provide stronger evidence on exposure and outcome.

In comparison to clinical trials, pharmacoepidemiological studies generally have larger sample size, more ethnic variety, longer follow-up duration, and reflect practice patterns in real world settings; however, selection bias in observational studies may confound the results, especially when study subjects have complicated comorbid conditions and multiple medications, as in schizophrenia patients. One of the best approaches to reduce biases is to use within-subject comparison, such as the case-series analysis with each subject himself as the control group [41] or the PSSA [18] in this study to assess antipsychotic associated AEs. These self-as-control methodologies allow for controlling of selection bias and unmeasured confounding factors.

Using a large nationwide sample was a strength of the current study, where the database represented the entire population of Taiwan. Because antipsychotics were reimbursed by NHI in Taiwan, all prescriptions in schizophrenia patients have been recorded in the NHIRD. Additionally, patients with catastrophic illness (including schizophrenia) as defined by Department of Health, Taiwan, are exempted from copayments. Therefore, access to medical care and adherence to antipsychotic regimens is less affected by economic burden in patients with catastrophic illness. This helps to ensure all medical care utilization data is captured completely. We included only new users in this study in an attempt to create a relatively homogenous cohort, and increase signal/noise ratio in the risk detection. The PSSA method in studying patients with schizophrenia was validated by referencing previous reports, e.g., the results of EPS risk evaluation were largely consistent with one meta-analysis [25]; the results of hyperprolactinemia in amisulpride [30], hyperglycemia in olanzapine [25], and cardiac arrhythmias in haloperidol [42] were also consistent with previous studies, thus lending more credibility to generalize our results. To our knowledge, this is the first comprehensive exploration of therapeutic risk of sulpiride, which complements previous literature on sulpiride effectiveness [5], suggesting avoidance of sulpiride for patients already at higher risk of AEs, and providing better information for personalized schizophrenia therapy strategy. Although sulpiride is not commonly used in Western countries, the present study provides supportive information to countries where this medication is still commonly prescribed in clinical practices.

A limitation of the current study is the exclusion of patients who switched between different antipsychotics. For example, patients with milder EPS might be switched to antipsychotics known to be less likely to induce EPS (e.g., quetiapine), rather than treated by drugs (e.g., trihexyphenidyl). Future cohort studies are necessary to accurately chronicle the exposure and outcome events in order to assess the impact of drug switching. Although one option to evaluate switching is to restrict the study subjects to only those without switching antipsychotics, as has been performed in a previous study [20], it is possible that such restriction might reduce sample size, statistical power, and generalizability of study results. We did not investigate the dose-response relationship between sulpiride and AEs in the study because only patients who had both the marker and index drugs were included in the analyses (case-only design). A cohort study could be considered to provide a threshold between therapeutic dosage and toxic concentration of sulpiride. Like most of administrative claims database, OTC drugs are not covered by NHIRD; we were unable to evaluate their associations with sulpiride in this study. Because patients with severe conditions, including schizophrenia, are most likely received prescription drugs rather than OTC drugs, the limitation of unable to trace OTC use might be negligible. Moreover, PSSA used in this study was based on within-individual comparisons; unless a patient changes his behavior in OTC use during the time period corresponding to this study, the impact of OTC use on study results could be minimal. We restricted the study period to 12 months before and after incident index drug use in current analyses to reduce potential impacts from within-subject confounding that could happen during a long-term study (e.g., change in diet and other behavior). The tradeoff is that, by limiting the study period to 1 year before and after the index event, we might have missed the opportunity to detect AEs that could develop only after long-term exposure.. Like all other study designs, PSSA also has the limitation in detecting low incidence AEs that can only be investigated with a study population of large sample size and/or long-term exposure and follow-up duration.

## Conclusion

We found sulpiride to be associated with an increased risk of EPS and hyperprolactinemia, and the potential risk could be as high as that induced by haloperidol and amisulpride, respectively. Additionally, our study provides grounds for future investigations into the associations between sulpiride and the increased use of additional drugs for managing adverse effects, including stomatological, dermatological, and musculoskeletal or joint side effects, constipation, and pneumonia.

## Supporting Information

File S1 Calculation of Null-effect Sequence Ratio. **Table S1 in File S1.** Exploratory analyses.
(DOCX)
