# Supplementary material for: Detecting Potential Adverse Reactions of Sulpiride in Schizophrenic Patients by Prescription Sequence Symmetry Analysis
Source: PLoS One. 2014 Feb 27;9(2):e89795. doi: 10.1371/journal.pone.0089795 (PMC3937342; doi:10.1371/journal.pone.0089795)
Supplement: File S1 — Calculation of Null-effect Sequence Ratio. Table S1 in File S1. Exploratory analyses. (DOCX) [file pone.0089795.s001.docx]

**File S1. Calculation of Null-effect Sequence Ratio**

This study, using a large nationwide database, attempted to provide information on therapeutic risk of antipsychotics. PSSA aims to test the propensity to initiate a marker drug (e.g., trihexyphenidyl) following the use of an index drug (e.g., antipsychotics), where the index drug is suspected to induce a side effect (e.g., extrapyramidal syndromes) that warrants treatment with the marker drug. The ratio of the patients initiating trihexyphenidyl after versus before antipsychotics initiation was described as the sequence ratio (SR):

$$Sequence ratio=\frac{Number of patients receiving \left( i \right) before (m)}{Number of patients receiving \left( m \right) before \left( i \right)},or\frac{\mathrm{Sequence}(i)\to(m)}{\mathrm{Sequence}(m)\to(i)}$$

Where, *(i)* indicated the index drug*,* and *(m)* indicated the marker drug.

Theoretically, if there is no causal relationship between the index and marker drugs, it is equally possible for a patient to initiate the marker drug before or after the initiation of the index drug; therefore, a symmetrical (or random) prescribing pattern of the marker drug should be observed around the initiation of the index drug. Conversely, if the index drug increases the risk of an adverse effect that requires treatment with the marker drug, it is expected that the marker drug is more likely to be initiated after, rather than before the index drug, leading to an asymmetrical prescribing pattern of the marker drug.

Due to the nature of within-subject comparison, PSSA and SR help to minimize the potential differences in baseline characteristics or those unrecognized confounding factors that could be present in between-subject comparisons. However, PSSA could be sensitive to prescribing trends over time; e.g., an event that makes the requirement of marker drugs grow rapidly. For this reason, the null-effect SR (*SR_null_*) was calculated in order to adjust for any possible temporal trends. *SR_null_* is the expected SR of an incidence trend when there is no causal relationship between the index and marker drugs, which provides a background rate for the chronological sequence of the two drugs. In this study, we computed the probability of antipsychotics→marker drug sequence for each antipsychotics user when the antipsychotic was first prescribed. Both the sequence *(i)→(m)* and the sequence *(m)→(i)* could also result from natural causes without any relation between the index drug and the marker drug. Thus we proposed to disaggregate the SR into several parts as:

$$SR=\frac{E_{i}}{E_{m}}\times B_{i\to m}$$

Where,

*E_i_* indicates the *exposure effect* of the index drug (the effect that results from the index drug does increase the risk of an adverse effect that requires treatment with the marker drug).

*E_m_* indicates the *exposure effect* of the marker drug (the effect that results from the marker drug does increase the risk of an adverse effect that requires treatment with the index drug).

*B_i→m_* indicates the *background rate* of the sequence *(i)→(m)*.

If there is no trend in time variance between the index and marker drugs (that is, the prescribing temporal trends are constant with time), *B_i→m_* should be equal to 1 and can be canceled out; however, the likelihood that trends change with time should be considered. *SR_null_* is calculated to be an adjusting factor for prescribing temporal trends. As described by *Hallas^[1](#_ENREF_1)^*, the *SR_null_* is an expected SR in the absence of any causal association, taking the incidence trends into account, which indicates the *background rate* for the prescribing sequence. *SR_null_* is derived from the calculation of probability, *P*, of each incident index drug user being exposed to a marker drug within the study period for his/her index drug-given day. Then the overall average probability, *P_a_*, is generated by weighting the number of incident users of an index drug on the drug-given date and averaging for all days, as:

$$P=\frac{\sum_{n=x+1}^{x+365} M_{n}}{\sum_{n=x-365}^{x-1} M_{n}+\sum_{n=x+1}^{x+365} M_{n}}$$

$$Pa=\frac{\sum_{n=1}^{u} [I_{n}\times(\sum_{n=x+1}^{x+365} M_{n})]}{\sum_{n=1}^{u} {[I}_{n}\times(\sum_{n=x-365}^{x-1} M_{n}+\sum_{n=x+1}^{x+365} M_{n})]}$$

Where,

*n* or *x* indicates consecutive days of the study period

*u* indicates the last day of the study period

*M_n_* indicates the number of persons receiving their first marker drug on the date.

*I_n_* indicates the number of persons receiving their first index drug on the date.

Finally, *SR_null_* is described as:

${SR}_{null}=\frac{P_{a}}{1-P_{a}}$

SR*_null_* can be regarded as an adjusting factor for the *background rate* of the two drugs prescribing sequence, thus *B_i→m_* can be canceled out while the SR*_null_* has been considered simultaneously:

$$B_{i\to m}\times\frac{1}{{SR}_{null}}=1$$

Summarily, the adjusted SR was described as:

$$Adjusted SR=\frac{E_{i}}{E_{m}}\times B_{i\to m}\times\frac{1}{{SR}_{null}}=\frac{E_{i}}{E_{m}}$$

If there is no association of sequence *(m)→(i)*, where the marker drug does not induce any side effect that needs to be treated with an index drug, $E_{m}=1$ $E_{m}=1$, the adjusted SR is indicated as the sole *exposure effect* of the index drug:

$Adjusted SR{=E}_{i}$

| **Table S1.** Exploratory analyses | | | | | | | |
| --- | --- | --- | --- | --- | --- | --- | --- |
| Drug classification | ATC | Causal/  noncausal | | | Sequence ratio | | |
|  |  |  |  |  | Crude | Adjusted | (95% CI) |
| Stomatological preparations | A01A | 48 | / | 23 | 2.09 | 1.86 | (1.13-3.07)* |
| Antacids | A02A | 199 | / | 582 | 0.34 | 0.46 | (0.39-0.54) |
| Drugs for peptic ulcer and gastro-oesophageal reflux disease | A02B | 150 | / | 190 | 0.79 | 0.84 | (0.68-1.04) |
| Other drugs for acid related disorders | A02X | 18 | / | 5 | 3.60 | 2.39 | (0.89-6.43) |
| Drugs for functional bowel disorders | A03A | 234 | / | 326 | 0.72 | 0.79 | (0.67-0.93) |
| Belladonna and derivatives, plain | A03B | 121 | / | 144 | 0.84 | 0.89 | (0.70-1.13) |
| Antispasmodics in combination with psycholeptics | A03C | 70 | / | 79 | 0.89 | 0.89 | (0.64-1.23) |
| Antispasmodics in combination with analgesics | A03D | 0 | / | 0 | NA | NA | ( . - . ) |
| Antispasmodics and anticholinergics in combination with other drugs | A03E | 12 | / | 26 | 0.46 | 0.53 | (0.27-1.05) |
| Propulsives | A03F | 204 | / | 249 | 0.82 | 0.86 | (0.72-1.04) |
| Antiemetics and antinauseants | A04A | 85 | / | 73 | 1.16 | 1.16 | (0.85-1.58) |
| Bile therapy | A05A | 6 | / | 2 | 3.00 | 2.33 | (0.47-11.6) |
| Liver therapy, lipotropics | A05B | 28 | / | 25 | 1.12 | 1.14 | (0.66-1.95) |
| Drugs for bile therapy and lipotropics in combination | A05C | 0 | / | 0 | NA | NA | ( . - . ) |
| Intestinal antiinfectives | A07A | 32 | / | 27 | 1.19 | 1.18 | (0.71-1.98) |
| Intestinal adsorbents | A07B | 56 | / | 50 | 1.12 | 1.12 | (0.76-1.64) |
| Electrolytes with carbohydrates | A07C | 1 | / | 0 | NA | NA | ( . - . ) |
| Antipropulsives | A07D | 106 | / | 106 | 1.00 | 1.02 | (0.78-1.34) |
| Intestinal antiinflammatory agents | A07E | 1 | / | 2 | 0.50 | 0.63 | (0.06-6.89) |
| Antidiarrheal microorganisms | A07F | 46 | / | 43 | 1.07 | 1.06 | (0.70-1.61) |
| Other antidiarrheals | A07X | 65 | / | 49 | 1.33 | 1.24 | (0.85-1.79) |
| Antiobesity preparations, excl. Diet products | A08A |  | / |  | NA | NA | ( . - . ) |
| Digestives, incl. Enzymes | A09A | 79 | / | 71 | 1.11 | 1.12 | (0.81-1.55) |
| Insulins and analogues | A10A | 11 | / | 10 | 1.10 | 1.22 | (0.52-2.88) |
| Blood glucose lowering drugs, excl. Insulins | A10B | 15 | / | 39 | 0.38 | 0.50 | (0.27-0.90) |
| Other drugs used in diabetes | A10X | 0 | / | 0 | NA | NA | ( . - . ) |
| Multivitamins, combinations | A11A | 3 | / | 9 | 0.33 | 0.69 | (0.19-2.54) |
| Multivitamins, plain | A11B | 1 | / | 1 | 1.00 | 1.00 | (0.06-16.0) |
| Vitamin a and d, incl. Combinations of the two | A11C | 1 | / | 1 | 1.00 | 1.00 | (0.06-16.0) |
| Vitamin b1, plain and in combination with vitamin b6 and b12 | A11D | 41 | / | 38 | 1.08 | 1.12 | (0.72-1.75) |
| Vitamin b-complex, incl. Combinations | A11E | 50 | / | 49 | 1.02 | 1.06 | (0.71-1.57) |
| Ascorbic acid (vitamin c), incl. Combinations | A11G | 10 | / | 10 | 1.00 | 1.06 | (0.44-2.55) |
| Other plain vitamin preparations | A11H | 39 | / | 24 | 1.63 | 1.51 | (0.91-2.51) |
| Other vitamin products, combinations | A11J | 77 | / | 58 | 1.33 | 1.27 | (0.90-1.79) |
| Calcium | A12A | 17 | / | 13 | 1.31 | 1.24 | (0.60-2.55) |
| **Table S1. (**Continued) | | | | | | | |
| Drug classification | ATC | Causal/  noncausal | | | Sequence ratio | | |
|  |  |  |  |  | Crude | Adjusted | (95% CI) |
| Potassium | A12B | 32 | / | 22 | 1.45 | 1.32 | (0.77-2.28) |
| Other mineral supplements | A12C | 3 | / | 3 | 1.00 | 0.89 | (0.18-4.43) |
| Tonics | A13A | 0 | / | 0 | NA | NA | ( . - . ) |
| Anabolic steroids | A14A | 0 | / | 0 | NA | NA | ( . - . ) |
| Other anabolic agents | A14B | 0 | / | 0 | NA | NA | ( . - . ) |
| Other alimentary tract and metabolism products | A16A | 4 | / | 4 | 1.00 | 0.94 | (0.23-3.76) |
| Antithrombotic agents | B01A | 51 | / | 58 | 0.88 | 0.94 | (0.65-1.37) |
| Antifibrinolytics | B02A | 74 | / | 70 | 1.06 | 1.06 | (0.76-1.47) |
| Vitamin K and other hemostatics | B02B | 10 | / | 8 | 1.25 | 1.09 | (0.43-2.76) |
| Iron preparations | B03A | 25 | / | 18 | 1.39 | 1.25 | (0.68-2.29) |
| Vitamin B12 and folic acid | B03B | 33 | / | 28 | 1.18 | 1.16 | (0.70-1.93) |
| Other antianemic preparations | B03X | 1 | / | 2 | 0.50 | 1.00 | (0.09-11.0) |
| Blood and related products | B05A | 20 | / | 8 | 2.50 | 2.08 | (0.92-4.72) |
| I.V. solutions | B05B | 134 | / | 134 | 1.00 | 1.01 | (0.80-1.28) |
| Irrigating solutions | B05C | 6 | / | 2 | 3.00 | 2.49 | (0.50-12.3) |
| Peritoneal dialytics | B05D | 0 | / | 1 | NA | NA | ( . - . ) |
| I.V. solution additives | B05X | 167 | / | 185 | 0.90 | 0.92 | (0.75-1.14) |
| Hemodialytics and hemofiltrates | B05Z | 0 | / | 0 | NA | NA | ( . - . ) |
| Other hematological agents | B06A | 163 | / | 175 | 0.93 | 0.98 | (0.80-1.22) |
| Cardiac glycosides | C01A | 3 | / | 4 | 0.75 | 0.85 | (0.19-3.79) |
| Cardiac stimulants excl. Cardiac glycosides | C01C | 19 | / | 10 | 1.90 | 1.73 | (0.81-3.72) |
| Vasodilators used in cardiac diseases | C01D | 22 | / | 26 | 0.85 | 0.88 | (0.50-1.55) |
| Other cardiac preparations | C01E | 1 | / | 3 | 0.33 | 0.43 | (0.04-4.12) |
| Antiadrenergic agents, centrally acting | C02A | 3 | / | 0 | NA | NA | ( . - . ) |
| Antiadrenergic agents, ganglion-blocking | C02B | 0 | / | 0 | NA | NA | ( . - . ) |
| Antiadrenergic agents, peripherally acting | C02C | 5 | / | 17 | 0.29 | 0.40 | (0.15-1.08) |
| Arteriolar smooth muscle, agents acting on | C02D | 3 | / | 4 | 0.75 | 0.66 | (0.15-2.96) |
| Other antihypertensives | C02K | 0 | / | 0 | NA | NA | ( . - . ) |
| Antihypertensives and diuretics in combination | C02L | 8 | / | 9 | 0.89 | 0.88 | (0.34-2.29) |
| Combinations of antihypertensives in atc-gr. C02 | C02N | 0 | / | 0 | NA | NA | ( . - . ) |
| Low-ceiling diuretics, thiazides | C03A | 15 | / | 11 | 1.36 | 1.34 | (0.62-2.92) |
| Low-ceiling diuretics, excl. Thiazides | C03B | 9 | / | 10 | 0.90 | 0.96 | (0.39-2.35) |
| High-ceiling diuretics | C03C | 40 | / | 27 | 1.48 | 1.42 | (0.87-2.31) |
| Potassium-sparing agents | C03D | 10 | / | 4 | 2.50 | 1.95 | (0.61-6.23) |
| Diuretics and potassium-sparing agents in combination | C03E | 9 | / | 4 | 2.25 | 1.80 | (0.55-5.83) |
| **Table S1. (**Continued) | | | | | | | |
| Drug classification | ATC | Causal/  noncausal | | | Sequence ratio | | |
|  |  |  |  |  | Crude | Adjusted | (95% CI) |
| Other diuretics | C03X | 0 | / | 0 | NA | NA | ( . - . ) |
| Peripheral vasodilators | C04A | 80 | / | 90 | 0.89 | 0.93 | (0.69-1.25) |
| Agents for treatment of hemorrhoids and anal fissures | C05A | 26 | / | 21 | 1.24 | 1.21 | (0.68-2.14) |
| Agents for treatment of hemorrhoids and anal fissures | C05A | 26 | / | 21 | 1.24 | 1.21 | (0.68-2.14) |
| Antivaricose therapy | C05B | 15 | / | 9 | 1.67 | 1.64 | (0.72-3.76) |
| Capillary stabilizing agents | C05C | 10 | / | 8 | 1.25 | 1.28 | (0.51-3.25) |
| Beta blocking agents | C07A | 225 | / | 146 | 1.54 | 1.42 | (1.12-1.71)* |
| Beta blocking agents and thiazides | C07B | 0 | / | 0 | NA | NA | ( . - . ) |
| Beta blocking agents and other diuretics | C07C | 2 | / | 2 | 1.00 | 0.78 | (0.11-5.52) |
| Beta blocking agents, thiazides and other diuretics | C07D | 0 | / | 0 | NA | NA | ( . - . ) |
| Beta blocking agents and vasodilators | C07E | 0 | / | 0 | NA | NA | ( . - . ) |
| Beta blocking agents and other antihypertensives | C07F | 0 | / | 0 | NA | NA | ( . - . ) |
| Selective calcium channel blockers with mainly vascular effects | C08C | 39 | / | 50 | 0.78 | 0.81 | (0.54-1.24) |
| Selective calcium channel blockers with direct cardiac effects | C08D | 8 | / | 15 | 0.53 | 0.68 | (0.29-1.61) |
| Non-selective calcium channel blockers | C08E | 0 | / | 0 | NA | NA | ( . - . ) |
| Calcium channel blockers and diuretics | C08G | 0 | / | 0 | NA | NA | ( . - . ) |
| Ace inhibitors, plain | C09A | 22 | / | 31 | 0.71 | 0.80 | (0.46-1.39) |
| Ace inhibitors, combinations | C09B | 0 | / | 0 | NA | NA | ( . - . ) |
| Angiotensin ii antagonists, plain | C09C | 11 | / | 17 | 0.65 | 0.66 | (0.31-1.42) |
| Angiotensin ii antagonists, combinations | C09D | 3 | / | 4 | 0.75 | 0.72 | (0.16-3.22) |
| Other agents acting on the renin-angiotensin system | C09X | 0 | / | 0 | NA | NA | ( . - . ) |
| Lipid modifying agents, plain | C10A | 26 | / | 32 | 0.81 | 0.93 | (0.55-1.55) |
| Lipid modifying agents, combinations | C10B | 0 | / | 2 | 0.00 | 0.00 | ( . - . ) |
| Antifungals for topical use | D01A | 92 | / | 75 | 1.23 | 1.19 | (0.88-1.62) |
| Antifungals for systemic use | D01B | 11 | / | 4 | 2.75 | 1.90 | (0.61-5.97) |
| Emollients and protectives | D02A | 38 | / | 28 | 1.36 | 1.35 | (0.83-2.19) |
| Protectives against uv-radiation | D02B | 0 | / | 0 | NA | NA | ( . - . ) |
| Cicatrizants | D03A | 2 | / | 1 | 2.00 | 1.60 | (0.15-17.6) |
| Enzymes | D03B | 0 | / | 0 | NA | NA | ( . - . ) |
| Antipruritics, incl. Antihistamines, anesthetics, etc. | D04A | 33 | / | 17 | 1.94 | 1.61 | (0.89-2.88) |
| Antipsoriatics for topical use | D05A | 7 | / | 6 | 1.17 | 1.24 | (0.42-3.68) |
| Antipsoriatics for systemic use | D05B | 0 | / | 0 | NA | NA | ( . - . ) |
| Antibiotics for topical use | D06A | 92 | / | 69 | 1.33 | 1.28 | (0.93-1.75) |
| Chemotherapeutics for topical use | D06B | 14 | / | 8 | 1.75 | 1.58 | (0.66-3.78) |
| Antibiotics and chemotherapeutics, combinations | D06C | 0 | / | 0 | NA | NA | ( . - . ) |
| **Table S1. (**Continued) | | | | | | | |
| Drug classification | ATC | Causal/  noncausal | | | Sequence ratio | | |
|  |  |  |  |  | Crude | Adjusted | (95% CI) |
| Corticosteroids, plain | D07A | 145 | / | 143 | 1.01 | 1.04 | (0.83-1.31) |
| Corticosteroids, combinations with antiseptics | D07B | 3 | / | 1 | 3.00 | 1.80 | (0.19-17.3) |
| Corticosteroids, combinations with antibiotics | D07C | 125 | / | 135 | 0.93 | 0.98 | (0.77-1.25) |
| Corticosteroids, other combinations | D07X | 42 | / | 15 | 2.80 | 2.18 | (1.21-3.92) |
| Antiseptics and disinfectants | D08A | 16 | / | 8 | 2.00 | 1.81 | (0.78-4.24) |
| Medicated dressings | D09A | 0 | / | 0 | NA | NA | ( . - . ) |
| Anti-acne preparations for topical use | D10A | 52 | / | 43 | 1.21 | 1.24 | (0.83-1.86) |
| Anti-acne preparations for systemic use | D10B | 0 | / | 0 | NA | NA | ( . - . ) |
| Other dermatological preparations | D11A | 2 | / | 0 | NA | NA | ( . - . ) |
| Antiinfectives and antiseptics, excl. Combinations | G01A | 47 | / | 57 | 0.82 | 0.87 | (0.59-1.29) |
| Antiinfectives and antiseptics, excl. Combinations | G01A | 47 | / | 57 | 0.82 | 0.87 | (0.59-1.29) |
| Antiinfectives/antiseptics in combination with corticosteroids | G01B | 0 | / | 0 | NA | NA | ( . - . ) |
| Oxytocics | G02A | 12 | / | 24 | 0.50 | 0.58 | (0.29-1.16) |
| Contraceptives for topical use | G02B | 0 | / | 0 | NA | NA | ( . - . ) |
| Hormonal contraceptives for systemic use | G03A | 0 | / | 0 | NA | NA | ( . - . ) |
| Androgens | G03B | 4 | / | 0 | NA | NA | ( . - . ) |
| Estrogens | G03C | 35 | / | 26 | 1.35 | 1.35 | (0.81-2.25) |
| Progestogens | G03D | 58 | / | 42 | 1.38 | 1.31 | (0.88-1.95) |
| Androgens and female sex hormones in combination | G03E | 0 | / | 3 | NA | NA | ( . - . ) |
| Progestogens and estrogens in combination | G03F | 18 | / | 11 | 1.64 | 1.56 | (0.74-3.30) |
| Gonadotropins and other ovulation stimulants | G03G | 7 | / | 8 | 0.88 | 0.82 | (0.30-2.27) |
| Antiandrogens | G03H | 2 | / | 2 | 1.00 | 1.00 | (0.14-7.10) |
| Other sex hormones and modulators of the genital system | G03X | 0 | / | 1 | NA | NA | ( . - . ) |
| Other urologicals, incl. Antispasmodics | G04B | 61 | / | 56 | 1.09 | 1.14 | (0.79-1.63) |
| Drugs used in benign prostatic hypertrophy | G04C | 13 | / | 17 | 0.76 | 0.85 | (0.41-1.75) |
| Anterior pituitary lobe hormones and analogues | H01A | 0 | / | 0 | NA | NA | ( . - . ) |
| Posterior pituitary lobe hormones | H01B | 7 | / | 8 | 0.88 | 0.84 | (0.30-2.31) |
| Hypothalamic hormones | H01C | 0 | / | 0 | NA | NA | ( . - . ) |
| Corticosteroids for systemic use, plain | H02A | 159 | / | 188 | 0.85 | 0.92 | (0.74-1.13) |
| Corticosteroids for systemic use, combinations | H02B | 0 | / | 0 | NA | NA | ( . - . ) |
| Antiadrenal preparations | H02C | 0 | / | 0 | NA | NA | ( . - . ) |
| Thyroid preparations | H03A | 4 | / | 3 | 1.33 | 1.39 | (0.31-6.21) |
| Antithyroid preparations | H03B | 5 | / | 1 | 5.00 | 4.00 | (0.47-34.2) |
| Iodine therapy | H03C | 1 | / | 1 | 1.00 | 3.00 | (0.19-48.0) |
| Glycogenolytic hormones | H04A | 0 | / | 0 | NA | NA | ( . - . ) |
| **Table S1. (**Continued) | | | | | | | |
| Drug classification | ATC | Causal/  noncausal | | | Sequence ratio | | |
|  |  |  |  |  | Crude | Adjusted | (95% CI) |
| Parathyroid hormones and analogues | H05A | 0 | / | 0 | NA | NA | ( . - . ) |
| Anti-parathyroid agents | H05B | 2 | / | 0 | NA | NA | ( . - . ) |
| Tetracyclines | J01A | 69 | / | 87 | 0.79 | 0.86 | (0.62-1.17) |
| Amphenicols | J01B | 26 | / | 27 | 0.96 | 1.01 | (0.59-1.73) |
| Beta-lactam antibacterials, penicillins | J01C | 221 | / | 229 | 0.97 | 1.01 | (0.84-1.21) |
| Other beta-lactam antibacterials | J01D | 232 | / | 269 | 0.86 | 0.90 | (0.76-1.07) |
| Sulfonamides and trimethoprim | J01E | 56 | / | 42 | 1.33 | 1.31 | (0.88-1.95) |
| Macrolides, lincosamides and streptogramins | J01F | 111 | / | 100 | 1.11 | 1.09 | (0.83-1.43) |
| Aminoglycoside antibacterials | J01G | 72 | / | 56 | 1.29 | 1.22 | (0.86-1.73) |
| Quinolone antibacterials | J01M | 62 | / | 39 | 1.59 | 1.50 | (1.00-2.24) |
| Combinations of antibacterials | J01R | 0 | / | 0 | NA | NA | ( . - . ) |
| Other antibacterials | J01X | 25 | / | 17 | 1.47 | 1.40 | (0.76-2.59) |
| Antimycotics for systemic use | J02A | 18 | / | 22 | 0.82 | 0.82 | (0.44-1.53) |
| Drugs for treatment of tuberculosis | J04A | 1 | / | 4 | 0.25 | 0.32 | (0.04-2.85) |
| Drugs for treatment of lepra | J04B | 0 | / | 0 | NA | NA | ( . - . ) |
| Direct acting antivirals | J05A | 5 | / | 2 | 2.50 | 2.04 | (0.40-10.5) |
| Immune sera | J06A | 0 | / | 0 | NA | NA | ( . - . ) |
| Immunoglobulins | J06B | 0 | / | 0 | NA | NA | ( . - . ) |
| Alkylating agents | L01A | 3 | / | 0 | NA | NA | ( . - . ) |
| Antimetabolites | L01B | 1 | / | 3 | 0.33 | 0.43 | (0.04-4.12) |
| Plant alkaloids and other natural products | L01C | 1 | / | 0 | NA | NA | ( . - . ) |
| Cytotoxic antibiotics and related substances | L01D | 3 | / | 0 | NA | NA | ( . - . ) |
| Other antineoplastic agents | L01X | 1 | / | 0 | NA | NA | ( . - . ) |
| Hormones and related agents | L02A | 1 | / | 4 | 0.25 | 0.32 | (0.04-2.85) |
| Hormone antagonists and related agents | L02B | 1 | / | 1 | 1.00 | 1.00 | (0.06-16.0) |
| Immunostimulants | L03A | 1 | / | 1 | 1.00 | 1.00 | (0.06-16.0) |
| Immunosuppressants | L04A | 3 | / | 0 | NA | NA | ( . - . ) |
| Antiinflammatory and antirheumatic products, non-steroids | M01A | 224 | / | 566 | 0.40 | 0.49 | (0.42-0.57) |
| Antiinflammatory/antirheumatic agents in combination | M01B | 0 | / | 0 | NA | NA | ( . - . ) |
| Specific antirheumatic agents | M01C | 0 | / | 0 | NA | NA | ( . - . ) |
| Topical products for joint and muscular pain | M02A | 103 | / | 77 | 1.34 | 1.31 | (0.97-1.76) |
| Muscle relaxants, peripherally acting agents | M03A | 5 | / | 1 | 5.00 | 2.83 | (0.33-24.2) |
| Muscle relaxants, centrally acting agents | M03B | 190 | / | 267 | 0.71 | 0.79 | (0.66-0.95) |
| Muscle relaxants, directly acting agents | M03C | 0 | / | 0 | NA | NA | ( . - . ) |
| Antigout preparations | M04A | 29 | / | 34 | 0.85 | 0.95 | (0.58-1.56) |
| **Table S1. (**Continued) | | | | | | | |
| Drug classification | ATC | Causal/  noncausal | | | Sequence ratio | | |
|  |  |  |  |  | Crude | Adjusted | (95% CI) |
| Drugs affecting bone structure and mineralization | M05B | 0 | / | 2 | 0.00 | 0.00 | ( . - . ) |
| Other drugs for disorders of the musculo-skeletal system | M09A | 0 | / | 0 | NA | NA | ( . - . ) |
| Agents against amoebiasis and other protozoal diseases | P01A | 22 | / | 29 | 0.76 | 0.77 | (0.44-1.34) |
| Antimalarials | P01B | 0 | / | 2 | 0.00 | 0.00 | ( . - . ) |
| Agents against leishmaniasis and trypanosomiasis | P01C | 0 | / | 0 | NA | NA | ( . - . ) |
| Antitrematodals | P02B | 0 | / | 0 | NA | NA | ( . - . ) |
| Antinematodal agents | P02C | 2 | / | 1 | 2.00 | 2.50 | (0.23-27.6) |
| Anticestodals | P02D | 0 | / | 0 | NA | NA | ( . - . ) |
| Ectoparasiticides, incl. Scabicides | P03A | 4 | / | 2 | 2.00 | 1.60 | (0.29-8.74) |
| Insecticides and repellents | P03B | 0 | / | 0 | NA | NA | ( . - . ) |
| Decongestants and other nasal preparations for topical use | R01A | 22 | / | 20 | 1.10 | 1.10 | (0.60-2.02) |
| Nasal decongestants for systemic use | R01B | 203 | / | 263 | 0.77 | 0.85 | (0.71-1.02) |
| Throat preparations | R02A | 1 | / | 0 | NA | NA | ( . - . ) |
| Adrenergics, inhalants | R03A | 18 | / | 20 | 0.90 | 0.93 | (0.49-1.75) |
| Other drugs for obstructive airway diseases, inhalants | R03B | 12 | / | 10 | 1.20 | 1.13 | (0.49-2.62) |
| Adrenergics for systemic use | R03C | 136 | / | 201 | 0.68 | 0.74 | (0.60-0.92) |
| Other systemic drugs for obstructive airway diseases | R03D | 155 | / | 176 | 0.88 | 0.92 | (0.74-1.14) |
| Expectorants, excl. Combinations with cough suppressants | R05C | 235 | / | 355 | 0.66 | 0.73 | (0.62-0.86) |
| Cough suppressants, excl. Combinations with expectorants | R05D | 219 | / | 399 | 0.55 | 0.64 | (0.54-0.75) |
| Cough suppressants and expectorants, combinations | R05F | 197 | / | 305 | 0.65 | 0.72 | (0.60-0.86) |
| Other cold preparations | R05X | 93 | / | 131 | 0.71 | 0.77 | (0.59-1.00) |
| Antihistamines for systemic use | R06A | 259 | / | 497 | 0.52 | 0.62 | (0.53-0.72) |
| Other respiratory system products | R07A | 0 | / | 0 | NA | NA | ( . - . ) |
| Antiinfectives | S01A | 107 | / | 164 | 0.65 | 0.73 | (0.57-0.93) |
| Antiinflammatory agents | S01B | 51 | / | 70 | 0.73 | 0.79 | (0.55-1.13) |
| Antiinflammatory agents and antiinfectives in combination | S01C | 74 | / | 80 | 0.93 | 0.95 | (0.70-1.31) |
| Antiglaucoma preparations and miotics | S01E | 53 | / | 48 | 1.10 | 1.06 | (0.72-1.57) |
| Mydriatics and cycloplegics | S01F | 10 | / | 3 | 3.33 | 1.93 | (0.53-7.02) |
| Decongestants and antiallergics | S01G | 23 | / | 31 | 0.74 | 0.82 | (0.48-1.40) |
| Local anesthetics | S01H | 2 | / | 4 | 0.50 | 0.63 | (0.11-3.41) |
| Diagnostic agents | S01J | 0 | / | 0 | NA | NA | ( . - . ) |
| Surgical aids | S01K | 9 | / | 14 | 0.64 | 0.70 | (0.30-1.62) |
| Ocular vascular disorder agents | S01L | 0 | / | 0 | NA | NA | ( . - . ) |
| Other ophthalmologicals | S01X | 41 | / | 40 | 1.03 | 1.08 | (0.70-1.67) |
| Antiinfectives | S02A | 7 | / | 4 | 1.75 | 1.36 | (0.40-4.66) |
| **Table S1. (**Continued) | | | | | | | |
| Drug classification | ATC | Causal/  noncausal | | | Sequence ratio | | |
|  |  |  |  |  | Crude | Adjusted | (95% CI) |
| Corticosteroids | S02B | 0 | / | 0 | NA | NA | ( . - . ) |
| Corticosteroids and antiinfectives in combination | S02C | 5 | / | 2 | 2.50 | 2.04 | (0.40-10.5) |
| Other otologicals | S02D | 1 | / | 1 | 1.00 | 1.00 | (0.06-16.0) |
| Antiinfectives | S03A | 0 | / | 0 | NA | NA | ( . - . ) |
| Corticosteroids | S03B | 0 | / | 0 | NA | NA | ( . - . ) |
| Corticosteroids and antiinfectives in combination | S03C | 0 | / | 0 | NA | NA | ( . - . ) |
| Other ophthalmological and otological preparations | S03D | 0 | / | 0 | NA | NA | ( . - . ) |
| *Statistical significant at alpha level at 0.05.  ^†^Anatomical Therapeutic Chemical (ATC) classification system developed by WHO Collaborating Centre  ^‡^NA: Not applicable | | | | | | | |
